# Identification of key somatic features that are common and the ones that differ between swim strokes through allometric modeling

**DOI:** 10.3389/fspor.2023.1308033

**Published:** 2023-12-01

**Authors:** Marek Rejman, Alan M. Nevill, Nuno D. Garrido, Daria Rudnik, Jorge E. Morais

**Affiliations:** ^1^ Faculty of Physical Education and Sport, Wroclaw University of Health and Sport Sciences, Wroclaw, Poland; ^2^ Faculty of Education, Health, and Wellbeing, University of Wolverhampton, Wolverhampton, United Kingdom; ^3^ Department of Sport Sciences, University of Trás-os-Montes and Alto Douro, Vila Real, Portugal; ^4^ Research Centre in Sports, Health and Human Development (CIDESD), Vila Real, Portugal; ^5^ Department of Sport Sciences, Polytechnic Institute of Bragança, Bragança, Portugal

**Keywords:** swimming, anthropometrics, body dimensions, performance, modeling

## Abstract

**Introduction:**

The aim of this study was to explore which key somatic features are common to four swim strokes and medley, and specifically to identify which characteristics benefit only specific strokes.

**Methods:**

The sample was composed of 130 swimmers (95 males aged 19.5 ± 2.9 years and 35 females aged 18.4 ± 2.8 years). A set of anthropometric variables was used to predict swimming speed in the four swimming strokes and medley.

**Results:**

A multiplicative model with allometric body size components was used to identify the demographic and anthropometric predictors of swimming speed. Trunk height and waist circumference were the only variables significantly different among swimming strokes (*p* < 0.05). Associations between swimming speed and arm length were similar in breaststroke and medley, and in freestyle, backstroke and butterfly (*R*^2^ = 60.9%). The model retained as swimming speed predictors the age^2^, upper body circumference, hand breadth, waist circumference, and subscapular skinfold thickness (these last two had negative associations).

**Conclusion:**

All these predictors were common to all four swim strokes and medley. Arm length was also retained as a significant predictor, but this one varied significantly between the four different swim strokes and medley. These findings highlight the importance of having a “V-shape” trunk, longer upper limbs, and large hands as predictors of swimming performance.

## 1. Introduction

Sports performance is strongly determined by body features (1, 2). The same occurs in competitive swimming. Studies reported that swimmers who have bigger somatic features (specifically, limbs’ lengths, girths, and hand surface area) are more likely to deliver better performances (3, 4).

Notwithstanding, most research about this topic is related to the front-crawl stroke. Being the fastest stroke (5), researchers put a lot of focus in this swim stroke. Overall, the better performing swimmers in front-crawl are taller, bigger, with a larger arm span, and greater hand and foot surface areas (6, 7). By contrast, scarce evidence can be found in the remaining swim strokes (backstroke, breaststroke, and butterfly) (8), and even less in medley (9). Nonetheless, for young male and female breaststrokers aged between 10 and 13 years, it was found that the forearm girth and the leg length were (among others) the strongest predictors of the 100 m breaststroke event with a positive association (i.e., larger dimensions led to fastest performances) (10). In backstroke, the same research group found out that the strongest predictors of the 100 m event were the sitting height (SH), leg length, and two girths (forearm and arm relaxed girth) (11). In butterfly, Dimitric et al. (12), noted that arm span in girls was significantly related with better performances in the 50 m event. As for medley, a study with young swimmers noted significant correlations between the 200 m medley event and height, hand length, arm span, and body fat percentage (this with a negative correlation) in boys. As for girls, non-significant correlations were found between anthropometrics and the 200 m medley event. Notwithstanding, all these findings are for sprinting events, i.e., 50 or 100 m (and middle-distance for the medley) and in youth swimming. Thus, the literature lacks information about the role that somatic features play on other race distances in all swim strokes and medley, and in older age groups.

Some of these outputs were based on allometric modeling (10, 11). This approach consists in fitting a straight line to logarithmic transformations of the original bivariate data and then back-transforming the resulting equation to form a two-parameter power function in the arithmetic scale. The method has the dual advantages of enabling investigators to fit statistical models that describe multiplicative growth while simultaneously addressing the multiplicative nature of residual variation in response variables (heteroscedasticity) (13). Moreover, applying these allometric models to adult swimmers by providing deeper insights about the somatic features than can have a meaningful effect on each swim stroke performance can also help youth swimming coaches on understanding their swimmers’ handicaps and strengths.

The current scientific knowledge indicating that not having “ideal” anthropometrics can compromise the swimmers’ energetics and ultimately their performance. Therefore, it was hypothesized that applying these allometric models to adult swimmers will provide deeper insights about the somatic features of swimmers allowing us to identify those of them that provide the determinate effect on performance in each swim stroke. With this tool, coaches and their swimmers will be able to understand the handicaps and strengths to be determined by their unique somatic potential. To support our hypothesis, the aim of this study was to explore which key somatic features are common to all four swim strokes and individual medley, and specifically to identify which characteristics benefit only specific strokes, i.e., that are “stroke specific.”

## 2. Material and methods

### 2.1. Participants

The sample was composed of 130 swimmers (95 males aged 19.5 ± 2.9 years and 35 females aged 18.4 ± 2.8 years). They were recruited from Polish, Norwegian, and Portuguese swimming teams. The swimmers were classified in accordance with their level of swimming proficiency, determined by the World Aquatics Scoring Points (WASP) (https://www.worldaquatics.com/swimming/points). Consequently, the personal best scores (on preferred stroke, distance, and course length) were also noted. The swimmers were divided by the swimming stroke preferred for competition. For each event, swimming speed (SS) was calculated based on the time spent to cover a given event's distance. The swimmers’ performance level in WASP in each event and their race speed by sex are presented in Table 1. Swimmers were classified as Tier 3–4 athletes (14).

**Table 1:** Descriptive statistics (mean ± 1 SD) of the swimmers’ performance level (WASP) and swimming speed by sex in each event.

Freestyle	Males		Females	
	WAPS	Speed (m/s)	WAPS	Speed (m/s)
50 m	716 ± 152	2.17 ± 0.15	571 ± 86	1.75 ± 0.08
100 m	655 ± 122	1.85 ± 0.14	719	1.78
200 m	763 ± 114	1.84 ± 0.09	734 ± 79	1.67 ± 0.14
400 m	710 ± 112	1.65 ± 0.12	689 ± 133	1.51 ± 0.09
800 m	800 ± 142	1.62 ± 0.15	624 ± 112	1.42 ± 0.08
1,500 m	790 ± 86	1.64 ± 0.06		
Backstroke				
50 m	791 ± 163	2.01 ± 0.24	861 ± 16	1.77 ± 0.01
100 m	777 ± 81	1.81 ± 0.14	760 ± 181	1.58 ± 0.13
200 m	813 ± 196	1.76 ± 0.14	700 ± 129	1.47 ± 0.05
Breaststroke				
50 m	725 ± 50	1.77 ± 0.06	721 ± 118	1.55 ± 0.11
100 m	714 ± 76	1.59 ± 0.09	745 ± 101	1.49 ± 0.08
200 m	777 ± 76	1.49 ± 0.05	789 ± 13	1.34 ± 0.01
Butterfly				
50 m	723 ± 106	2.03 ± 0.12	857	1.95
100 m	793 ± 173	1.87 ± 0.13		
200 m	750 ± 88	1.67 ± 0.07	693	1.48
Medley				
100 m			933	1.73
200 m	682 ± 67	1.56 ± 0.02	748	1.49
400 m	798 ± 62	1.57 ± 0.03	678	1.32

All procedures performed in studies involving human participants were in accordance with the ethical standards of the institutional and/or national research committee and with the 1964 Helsinki declaration and its later amendments or comparable ethical standards. The study was approved by the local Ethics Institutional Board at Wroclaw University of Health and Sport Sciences, Poland (reference number 015/AWF/2020). Written informed consent was obtained directly from all individual, adult participants included in the study.

### 2.2. Measurement procedure

A series of anthropometric measurements was taken for each swimmer. One trained anthropologist (with assistant person) performed all the measurement in line with the standards developed by the International Society for the Advancement of Kinanthropometry (ISAK) (15). Testing was carried out using the following instruments after its proper calibration: stadiometer, (GPM, Switzerland), scale, sliding caliper (GPM, Switzerland), spreading caliper (GPM, Switzerland), anthropometric non-stretchable tape, and skinfold calipers (Harpenden Instruments, UK). All the measures were in a standardized order, recorded twice (the mean scores were retained for the statistical analyses). To determine the measurement error, all the procedures were repeated for every 10th respondent (intraClass correlation coefficient (ICC) between measurements ranged between 0.91 and 0.96).

The body height (m) and body mass (BM) (kg) were assessed (to the nearest 0.1 cm and 0.1 kg) for each swimmer. The following trunk measurements were performed. Trunk height (TH, in cm) was taken as vertical measurement of the distance from the basis to the upper edge of the sternum. SH (in cm), when the participant sat on a measurement box with their back and buttocks touching the backboard of the stadiometer, knees directed straight ahead, arms and hands resting at their side, and head in the Frankfort horizontal plane; upper body circumference (UBC, in cm) was measured with the tape wrapped around the trunk horizontally at the edge of sternum. For the chest circumference (CC, in cm), the tape was snugged horizontally around the chest through the xiphoid. Waist circumference (WC, in cm) was measured at the end of several consecutive natural breaths, at the level parallel to the floor, midpoint between the top of the iliac crest and the lower margin of the last palpable rib in the midaxillary line. The upper limb was described by calculating the following measurements. The upper limb length (ULL, in cm) was measured by the distance between the acromial and stylion landmarks. Arm length (AL, in cm) was determined as the distance between the marked acromial and radiale landmarks. The forearm length (FL, in cm) was measured by calculating the distance between the radiale and stylion landmark. For the hand length (HL, in cm), the measure was taken as the shortest distance from the marked mid-stylion line to the dactylion. Hand breadth (HB, in cm) was measured by the distance between the most prominent point on the lateral aspect of the head of the second metacarpal and the most prominent point on the medial aspect of the head of the fifth metacarpal. Arm span (AS, in cm) measurement was the distance between fingertips (dactylion III) when the arms are outstretched. Foot length (FL, in cm) and foot breadth (FB, in cm) were also measured. The length was determined as the distance from the acropodium (i.e., the tip of the longest toe which may be the first or second phalanx) to the pternion (i.e., most posterior point on the calcaneus of the foot). The breadth was the distance between the most prominent point on the medial aspect of head of first metatarsal and the most prominent point on the lateral aspect of head of fifth metatarsal. Triceps (TST, in mm) and subscapular (SST, in mm) skinfold thickness (SFT) were also measured.

### 2.3. Model design

**Figure 1:**
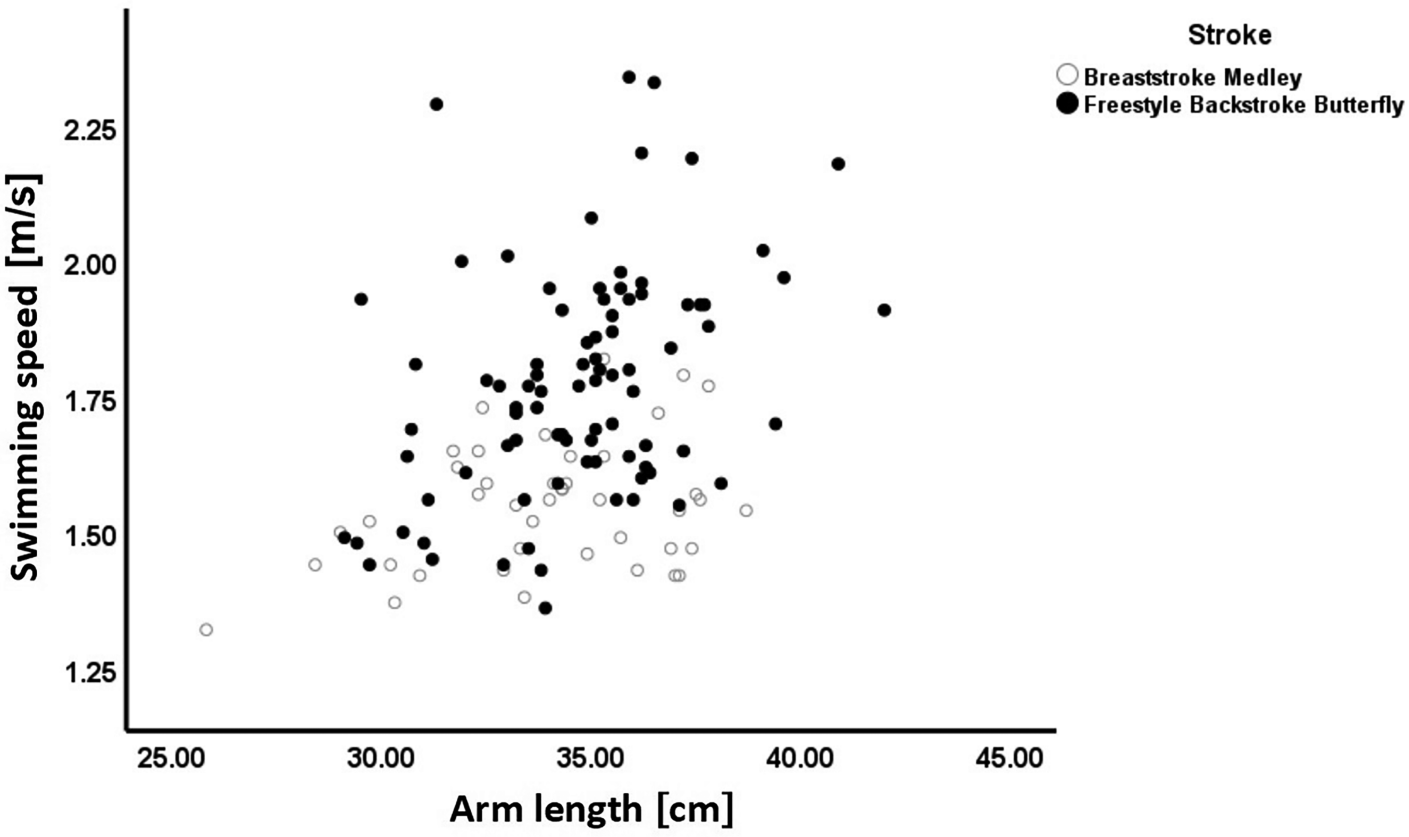
Relationship between swimming speed and arm length by swim stroke plus medley.

The mean and standard deviations were calculated as descriptive statistics. To identify the optimal demographic and somatic measurements, including BM, stature (H), skinfold thicknesses, and limb dimensions (lengths, breaths, and circumferences), associated with SS in all four swim strokes plus medley (four swim strokes combined) swimmers having controlled for age, we adopted the following multiplicative model with allometric body size components similar to those used to model the front-crawl swim speeds adopted by Nevill et al. (4, 16):

(1) $$\mathrm{SS}(m\cdot s-1)=a\cdot (\mathrm{BM})k1\cdot (H)k2\cdot \prod (\mathrm{LDi})\mathrm{ki}\cdot \exp(b\cdot \mathrm{age}+c\cdot \mathrm{ag}e^{2}+d\cdot \mathrm{SFT})\cdot \varepsilon ,$$

where “a” is a constant and Π (LDi) ki (i = 3, 4, 5, …) represents the product of limb segment dimensions raised to the power ki; with i = 3 being the TH, 4 = AS, 5 = SH, etc. (see list of variables in Table 2); and age, age^2^ and SFT, entered within an exponential term. This model has the advantages of having proportional body size components (see Figure 1) and the flexibility of a non-linear term in SFT and a quadratic in age, both within an exponential term that will ensure that the swim speeds will always remain non-negative irrespective of the swimmer's age and size of their SFT. Note that the multiplicative error ratio “*ε*” assumes that the error will increase in proportion to the swimmer's swim speed performance (display heteroscedastic errors).

**Table 2:** Descriptive statistics (mean ± SD) of the swimming performance, demographic and somatic measurements by stroke.

	Freestyle *N* = 58		Backstroke *N* = 15		Butterfly *N* = 14		Breaststroke *N* = 33		Medley *N* = 10		*p*-value
	Mean	SD	Mean	SD	Mean	SD	Mean	SD	Mean	SD	
Age	19.4	3.1	19.0	3.2	20.0	3.5	18.3	1.9	20.2	3.2	
BM (kg)	75.6	10.1	72.7	10.7	76.2	9.5	71.5	10.3	70.2	9.4	
H (cm)	182.1	7.8	179.0	8.6	177.6	7.5	179.2	9.2	176.6	7.1	
TH (cm)	149.3	6.7	146.4	7.1	143.9	6.9	146.2	7.8	144.8	6.1	0.037
AS (cm)	185.9	10.3	182.2	10.4	181.5	9.1	182.1	11.8	180.5	10.6	
SH (cm)	95.6	5.0	93.5	4.8	94.4	4.0	94.3	4.9	90.9	7.5	
UBC (cm)	112.8	8.1	109.6	8.2	114.8	7.6	110.0	7.9	108.7	7.3	
CC (cm)	92.6	7.7	89.1	7.6	92.7	5.4	88.7	6.6	88.7	8.8	
WC (cm)	78.6	5.8	76.1	6.1	79.3	3.1	74.9	6.3	76.5	5.4	0.032
TST (mm)	9.3	4.0	7.9	2.9	8.3	3.5	8.2	3.5	8.2	3.0	
SST (mm)	11.3	3.1	10.4	2.8	11.4	3.0	9.9	3.4	9.9	2.4	
HL (cm)	19.4	1.3	19.3	1.5	19.3	1.5	19.2	1.5	18.9	1.0	
HB (cm)	10.5	0.8	10.7	1.0	10.5	0.9	10.5	0.8	10.4	0.6	
FB (cm)	9.9	0.6	10.0	0.9	10.0	1.0	10.0	0.8	9.8	0.6	
FL (cm)	27.0	1.7	26.1	2.0	26.5	1.7	26.7	2.1	26.2	1.5	
AL (cm)	34.8	2.6	34.5	2.8	34.5	2.2	34.0	2.7	33.5	3.7	
FL (cm)	27.0	2.5	26.3	2.2	24.9	1.8	26.7	2.6	26.1	2.1	
ULL (cm)	81.5	4.7	81.2	4.9	79.0	4.4	79.9	5.6	78.6	5.2	

The model (Equation 1) can be linearized with a log transformation. A linear regression on Ln(SS) (ln = natural logarithms) can then be used to estimate the unknown parameters of the log-transformed model:

(2) $$\mathrm{Ln}(\mathrm{SS})=\ln(a)+k1\cdot \ln(\mathrm{BM})+k2\cdot \ln(H)+\sum \mathrm{ki}\cdot \ln(\mathrm{LDi})+b\cdot \mathrm{age}+c\cdot \mathrm{ag}e^{2}+d\cdot \mathrm{SFT}+\ln(\varepsilon ).$$

### 2.4. Statistical analysis

Having fitted the saturated model (all available demographic, somatic, and body size variables), an appropriate “parsimonious” model can be obtained using “backward elimination” (17) in which at each step the least important (non-significant) body size and limb segment dimensions variable is dropped from the current model. Further categorical or group differences within the population, e.g., swim stroke, can be explored by allowing the parameters in Equation 2 to vary for each group (by introducing them as fixed factors and associated interactions within an ANCOVA). The significance level was set at *p* < 0.05. Practical importance (meaningfulness) was assessed by reporting effect sizes (partial eta squared = *η*_p_^2^) as recommended by Winter et al. (18).

## 3. Results

Table 2 presents the descriptive statistics (mean ± standard deviation) of the swimmers’ age and somatic measurements by stroke. As a non-significant sex effect was noted in the following allometric model, data are presented with both sexes plotted together. The TH (0.037) and WC (0.032) were the only variables presenting a significant stroke effect, i.e., differences between strokes. In Figure 1, it is possible to note that associations between swimming speed and arm length are similar in breaststroke and medley, and in freestyle, backstroke and butterfly.

The parsimonious solution to the backward elimination regression analysis of log-transformed swim speed [Ln(SS)] resulted in the multiple regressions model presented in Table 3. The multiplicative allometric model relating swim speeds to the predictor variables found six variables that are significantly associated/predictors of Ln(SS) (note that the whole body size variables of BM and H were not significant and dropped from the analysis). Five of these predictors were found to be “common” to, or associated with, all five strokes. Three of these were positively associated with Ln(SS), which are Ln(UBC), Ln(HB), and age^2^. The other two “common” predictor variables were Ln(WC) and SST, both found to be negatively associated with Ln(SS) performance.

**Table 3:** The parsimonious solution to the backward elimination regression analysis to predict log-transformed swimming speed [Ln(SS)] given by Equation 2.

Parameter	B	SE	*t*	*p*-value	95% Confidence interval		*η*_p_^2^
					Lower bound	Upper bound	
Intercept	−1.553	0.547	−2.840	0.005	−2.635	−0.470	0.063
LN(UBC)	0.629	0.185	3.401	0.001	0.263	0.995	0.089
LN(HB)	0.813	0.343	2.368	0.019	0.133	1.492	0.045
LN(WC)	−1.012	0.380	−2.664	0.009	−1.765	−0.260	0.056
SST	−0.006	0.003	−2.434	0.016	−0.012	−0.001	0.047
Age^2^	0.0003	6.484 × 10^−05^	4.796	<0.001	0.0002	0.0004	0.162
Ln(AL)	0.037	0.275	0.135	0.893	−0.508	0.582	0.000
Ln(AL)_freestyle (Δ)	0.085	0.011	7.686	<0.001	0.063	0.106	0.332
Ln(AL)_backstroke (Δ)	0.075	0.016	4.815	<0.001	0.044	0.106	0.163
Ln(AL)_butterfly (Δ)	0.084	0.017	5.020	<0.001	0.051	0.117	0.175

*η*_p_^2^, partial eta squared.

The parameter estimates are means ± standard errors (SE) of estimate. Breaststroke and medley swimmers’ slope parameter for Ln(AL) was used as the baseline/reference parameter and the three other swim stroke parameters were compared with it, indicated by (Δ).

One other predictor variable, Ln(AL), was also found to be strongly associated with Ln(SS), but this association varied significantly between the four different swim strokes and medley (Table 3). This was identified by introducing stroke-by-predictor-variable interaction (see Statistical analysis section). The significant “stroke-by-arm length” interaction identified the arm length of freestyle (*p* < 0.001; *η*_p_^2 ^= 0.332), backstroke (*p* < 0.001; *η*_p_^2 ^= 0.163), and butterfly (*p* < 0.001; *η*_p_^2 ^= 0.175) swimmers to have a much stronger predictive ability on swim speed Ln(SS), compared to the baseline breaststroke and medley swimmer groups (*p* > 0.05) (see Table 3). The coefficient of determination, *R*^2^ for the fitted multiplicative allometric model was 60.9% with the log-transformed error ratio being 0.079 or 8.2%, having taken antilogs.

## 4. Discussion

The aim of this study was to explore which key somatic features are common to all four swim strokes and medley, and specifically to identify which characteristics benefit only specific strokes, i.e., that are “stroke specific.” Main findings revealed that associations between SS and AL were similar in breaststroke and medley, and in freestyle, backstroke and butterfly. The multiplicative allometric model found six variables significantly associated/predictors of Ln(SS) [(UBC), Ln(HB), Ln(WC), SST, age^2^, and Ln(AL)]. Ln(AL) was also found to be strongly associated with Ln(SS), but this association varied significantly between the four different swim strokes plus medley.

Since decades, the scientific papers strongly report the importance of anthropometric and somatic features in swimming performance (19). These are also pointed out as significant predictors in swimming performance talent identification programs allowing us to early identify which swimmers are more likely to perform better in when achieving adulthood (10, 20). As aforementioned, solid evidence can be found about the front-crawl stroke but less about the remaining swim strokes and medley. Moreover, and as far as our understanding goes, only one study included the four swim strokes aiming to identify which key somatic features are common to all of them, and which ones benefit only specific strokes, i.e., ones that are “stroke specific” (4). Notwithstanding, this was conducted with adolescent age-group swimmers. Our findings, including young adult national and international level swimmers (Table 3), revealed that Ln(UBC), Ln(HB), Ln(WC), SST, age^2^, and Ln(AL) were the predictors of SS. From these, Ln(UBC), Ln(HB), Ln(WC), SST, and age^2^ were common to all four swim strokes plus medley. Regarding the positive association of age^2^ with Ln(SS), swimming speed is rising within our swimmers’ age range (a quadratic within an exponential term). It was noted that elite swimmers are characterized by a high-level performance from 12 years on and progressively outperform swimmers from similar age (21). Thus, understanding this moment can help coaches and swimmers to better design and set realistic short- and long-term goals.

Regarding the predictors related to the swimmers’ trunk (Table 3), Ln(UBC) had a positive association and Ln(WC) a negative association. That is, greater UBC and smaller WC led to faster SS. At least in front-crawl, Papic et al. (22) aimed to determine the influence of torso morphology on maximum instantaneous hydrodynamic resistance. The authors noted that a larger area at the waist level was significantly correlated with a greater drag coefficient (greater drag leads to slower SS). Others, based on numerical simulations of male swimmers, analyzed the effect of different body shapes on hydrodynamic drag in the streamlined position (23). Four body shapes were scanned and analyzed: inverted triangle (“v-shape”), inverted trapezoid, rectangle, and oval shapes. From these, the inverted triangle was the one that presented the smallest projected area and surface area. Consequently, for speeds between 1.2 and 2.2 m/s, the inverted triangle shape was the one that presented smaller drag and drag coefficient values (23). This highlights the “v-shaped” body that swimmers have and allow them to achieve better performances. The SST also presented a negative association with SS, i.e., smaller thickness in the subscapular area led to faster SS. Overall, it was reported that a lean body mass and skinfolds thickness decrease were related to an enhancement of SS (24).

Ln(HB) was also retained as a significant predictor of Ln(SS) with a positive association with Ln(SS) (Table 3). Both experimental (25) and numerical studies (26) indicate the key role that the hand's dimensions (area, length, or width) have on the swimmers’ propulsion in front-crawl and, consequently, in the SS enhancement. The upper limbs, mainly the hands, are propelling surfaces that act as “paddles.” Thus, swimmers with larger hand areas are more likely to generate greater propulsive forces and hence faster SS (25). This rationale can be applied to the remaining swim strokes. Based on numerical simulations, it was possible to determine the hydrodynamic characteristics of the hands in various combinations of angle of attack and angle of orientation, as well as hand shape and speed, simulating the different phases of the stroke cycle in front-crawl (26). Based on four positions of the fingers (thumb adducted, thumb abducted, all fingers spread, and spreading fingers and thumb adducted), it was noted that at different moments within the stroke cycle, different finger positions delivered diverse propulsion outputs. Thus, swimmers can and should alter finger position and hand angles during the stroke cycle to generate more propulsion (26).

From the six predictors, Ln(AL) varied significantly between the four different swim strokes and medley with a positive association. Specifically, the arm length of freestylers, backstrokers, and butterflyers have a much stronger predictive ability on swim speed Ln(SS), compared to baseline breaststrokers and medley swimmers. Curiously, the three former swim strokes are the fastest ones and the two latter ones are the slowest (5). Biomechanically, SS can be improved by increasing stroke frequency, stroke length, or both (27). If stroke frequency can be trained or manipulated, stroke length is strongly correlated to the upper limbs’ length, namely, the arm span (28). It was found out that the arm length itself was a significant predictor of SS with a positive association in the front-crawl stroke (29). Moreover, swimmers with larger arm span (which includes the arm's length) are more likely to achieve greater distances with the hand entry and pull more water backward. This will lead to more propulsion and hence more SS. The arm span was one of the variables that was positively related to performance in all four swimming techniques and in individual medley (9). Nonetheless, our data revealed that longer upper limbs lengths (specifically the arm) are more important in the fastest swim strokes. Indeed, these (freestyle, butterfly, and backstroke) are more dependent from the SS achieved by the upper limbs rather than breaststroke (30).

Our findings demonstrate that it was possible to model SS in the four swim strokes plus medley based on allometric modeling. It should be mentioned that sex, swimmers’ level, and race distance were not retained as significant predictors. This means that the predictive model is suitable for both sexes, both competitive levels (i.e., national and elite), and all race distances. The five common predictors indicate that for all four swim strokes plus medley, there is a given moment in the swimmers’ career (age^2^), where performance may enhance exponentially, and a “v-shaped” trunk associated with a large hand area are significant predictors of SS. Swimmers with longer arms are more likely to deliver better performances in freestyle, butterfly, and backstroke. Based on these outputs (from older age groups where the growth likelihood is smaller), the practical applications become apparent—coaches of younger age-group swimmers must be aware of the importance that anthropometrics have on their swimmers’ performance. Due to the smaller number of swimmers, particularly in butterfly and medley, in comparison to the other strokes, the sample size can be considered as the main limitation of this study.

## 5. Conclusions

As a conclusion, allometric modeling indicated that five predictors were common to all swimmers—age^2^, UBC, HB, WC, and SST. These indicate that the large trunk “v-shape” morphology and the large hand areas are important factors in determining fast performances. One predictor (arm length) varied significantly between the four different swim strokes plus medley. This highlights the role that long upper limbs play in fastest swimming performance, particularly in freestyle, butterfly, and backstroke.

## Data Availability

The raw data supporting the conclusions of this article will be made available by the authors, without undue reservation.
